# Sezawa-Mode Surface Acoustic Wave Resonators in Pulsed-Laser-Deposited Pb_0.9_Ba_0.1_(Zr_0.52_,Ti_0.48_)O_3_ on Bulk Silicon

**DOI:** 10.3390/mi17070868

**Published:** 2026-07-22

**Authors:** Yves Janssens, Erwin Berenschot, Minh Nguyen, Niels Tas

**Affiliations:** MESA+ Institute and Faculty of Science and Technology, University of Twente, Drienerlolaan 5, 7522 NB Enschede, The Netherlandsj.w.berenschot@utwente.nl (E.B.); d.m.nguyen@utwente.nl (M.N.)

**Keywords:** Surface Acoustic Wave, resonator, PZT, silicon

## Abstract

Barium-doped Lead Zirconate Titanate Pb_0.9_Ba_0.1_(Zr_0.53_Ti_0.47_)O_3_ films with a (001)-dominant orientation were deposited on bulk silicon (Si) substrates using pulsed laser deposition (PLD). Due to the large electromechanical coupling coefficient (*K*^2^) of the P(B)ZT layer and the larger shear modulus of the Si substrate compared to the P(B)ZT film, it is possible to obtain higher-order acoustic-resonant modes (Sezawa mode) with SAW wavelength (*λ*)–piezoelectric film thickness (*h*) ratios below *h*/*λ* < 0.2. Due to the ferroelectric properties of the P(B)ZT film, the resonator’s performance can be improved by increasing the electric polarization. Consequently, the measured quality (*Q*) factors can be improved from 50 to 200 and the *K*^2^ values can be improved from 2 to 5% with the resonance frequency ranging from 275 to 500 MHz.

## 1. Introduction

Resonators based on Surface Acoustic Waves (SAWs) are widely applicable in many microelectromechanical system (MEMS) applications such as radio frequency (RF) filters [1], fully released resonators [2], sensing [3] and acoustofluidic actuation [4]. High quality factors (Q) and electromechanical coupling coefficients ($K^{2}$) are desired for these applications; therefore, single-crystal piezoelectric substrates such as LiNbO_3_ and LiTaO_3_ are commonly used. These substrates have two disadvantages: First, the low-phase velocities $V_{p}$, in the range of 3000–4000 m/s, which makes applications in the GHz range only possible with a sub-micron periodicity, p, if the interdigitated transducers (IDT) are to fit sufficiently small wavelengths, λ=2p. Secondly, the micromachining options of single-crystalline piezoelectric substrates are less developed, which limits the design possibilities for sensors, actuators and fully released resonators.

To overcome these issues, multilayered structures can be used in which the piezoelectric thin films are deposited onto single-crystalline substrates, e.g., silicon (Si) [5] and silicon carbide (SiC) [6,7]. The resonance frequency, $f=V_{p}/\lambda$, can be increased without decreasing the IDT periodicity when the piezoelectric thin films, e.g., AlN, ScAlN, GaN and ZnO, are deposited onto a substrate with a higher acoustic velocity than the piezoelectric film. However, none of these materials are ferroelectric. The hysteresis in ferroelectric materials is attractive because it allows for bias-dependent material properties which can be used for frequency or magnitude tuning [8,9]. Lead Zirconate Titanate (PZT) is both ferro- and piezoelectric and has a higher electromechanical coupling factor, $K^{2}$, and dielectric constant, $\epsilon_{r}$, than the piezoelectric materials commonly used for SAW applications. The $K^{2}$ of PZT is in the order of 20–35% [10], which is significantly higher than that of other piezoelectric materials, such as GaN (∼0.15%), ZnO (1.5–1.7%), and AlN (3.1–8%). Therefore, resonators made with PZT can be made smaller and large surface deformation can be obtained, making it an attractive material for specific RF-MEMS applications [9,11,12]. On the other hand, PZT has a low $V_{p}∼$ 1790 m/s [13], and can have high acoustic loss, lowering the Q-factor. Care has to be taken to mitigate the high parasitic capacitance [14,15], as a result of the high dielectric constant.

In this work, a proof of concept for the integration of barium (Ba)-doped PZT films deposited by pulsed laser deposition (PLD) onto bulk Si substrates is presented. PLD was chosen as the deposition method because of its good control over film composition and epitaxial characteristics, which result in better control over film crystallinity and preferential orientation, as compared to sputtering and sol-gel deposition [16]. An additional advantage of PLD is that high-quality piezoelectric films can be deposited in a single-temperature process (even down to CMOS-compatible temperatures [17]), whereas sol-gel processing typically requires a sequence of sintering and annealing steps, often up to 700 °C, to remove carbon-containing precursor residuals and crystallize the material in the desired crystal phase, often with less preferential orientation [18]. Excellent results can also be obtained with magnetron sputtering; however, this method typically gives amorphous films, when deposited at low temperature, and still requires a high-temperature annealing step to achieve the desired crystallinity [19].

The focus is on demonstrating the feasibility of combining the large piezoelectric coefficients and ferroelectric properties of PZT with the higher stiffness and processing advantages of silicon, enabling monolithic integration of actuation and sensing functionalities on a single platform. By implementing a SAW mode that penetrates relatively deep into the substrate, a high phase velocity can be realized (making use of the high shear velocity of Si), despite using PZT as the piezoelectric layer. By achieving a (001)-dominant orientation, optimizing the film thickness-to-wavelength ratio (h/λ<0.2) and piezoelectric domain reorientation by using a polarization voltage, the acoustic energy can be efficiently confined near the surface while maintaining a sufficiently high electromechanical coupling factor ($K^{2}$). Such integration not only enhances the performance of SAW-based resonators but also establishes a versatile platform for the design and fabrication of piezoelectric MEMS devices which require localized actuation and sensitive detection [20,21]. The results presented here serve as a first experimental step toward scalable, PZT-on-silicon systems, typically for advanced acoustoelectric and acoustofluidic applications.

## 2. Device Design and Modeling

**Figure 1:**
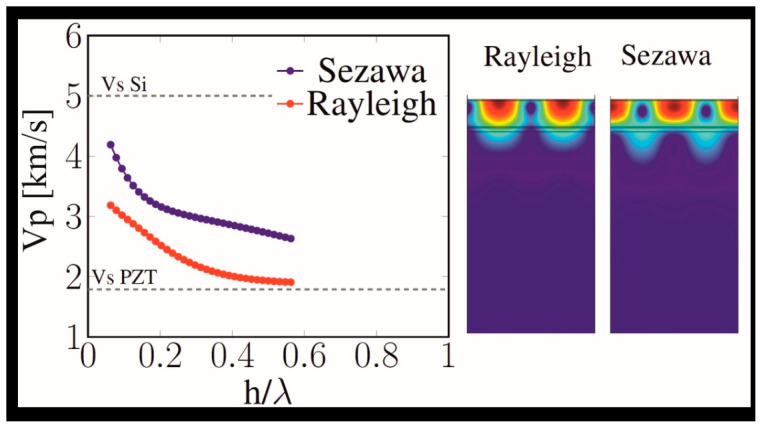
Dispersion relation analysis showing the increased phase velocity of the Sezawa mode and the corresponding displacement field for both modes. Note the deeper penetration of the displacement field into the substrate for the Sezawa mode. The shear velocities of pure silicon and PZT are indicated as *V*_s,Si_ and *V*_s,PZT_, respectively.

In order to define the resonance modes with the highest phase velocity and sufficiently high coupling coefficients, the SAW modes must be well defined in the surface resonant cavity, i.e., no bulk acoustic propagation modes. Therefore, the dispersions relations for the PZT multilayer are first determined to restrict the propagation modes close to the surface of the wafer. Typically, the resonant cavity for SAW consists of many IDTs with reflecting gratings placed along the direction of wave propagation. Part of the SAW is reflected on each grating and constructive interference is obtained when the reflected waves are in phase. In order to define a numerical model for determining the possible mode shapes, the total resonator is divided into multiple periodic unit cells where the width of the unit cell is equal to the SAW wavelength. To impose translation symmetry in the propagation direction the left and right boundaries of the unit cell are defined as periodic boundary conditions as $u\left(x=0\right)=u\left(x=\lambda \right)$. This condition states that the displacement field on the boundaries is equal. A two-dimensional finite element analysis is performed on the unit cell in COMSOL Multiphysics© and solved on a Cartesian grid. The stimulation domain consists of a silicon substrate with a thickness used in simulation of 10λ. The multilayer consists of 500 nm silicon oxide, 150 nm platinum and PZT with variable thickness. The 150 nm platinum IDT’s placed on top of the PZT layer have a width equal to λ/4. The simulated element consists of a single IDT, but the periodic boundary conditions make the simulated structure an infinitely long translationally invariant structure. The resulting dispersion curves determined by frequency domain analysis are shown in Figure 1. The solution is obtained for a fixed λ of 8 μm and variable thickness, h. The shear velocity for silicon ($V_{s,\mathrm{Si}}$) and PZT ($V_{s,\mathrm{PZT}}$) is indicated by dashed lines. From the dispersion curves, it is observed that no bulk acoustic modes propagate into the silicon substrate. Two modes are shown, the Rayleigh mode (symmetric mode) and the Sezawa mode (anti-symmetric mode); the latter is propagating with a higher phase velocity because of the higher shear modulus of the silicon substrate compared to the PZT layer.

The result is that the silicon substrate has a wave-guiding effect, as can be observed from the Sezawa mode shape. Compared with the Rayleigh mode, which is confined in the PZT layer, the Sezawa mode penetrates deeper into the multilayer structure. As the thickness of the PZT layer is increased, the phase velocity of the two modes converges to the shear velocity of PZT. The numerical results show that, supported by the relative strong electromechanical couple in PZT, SAW propagation for both modes is possible even for normalized thickness h/λ<0.1. The relatively high phase velocity for thin PZT-on-Si Sezawa modes is important for reducing fabrication complexity for high-frequency devices.

**Figure 2:**
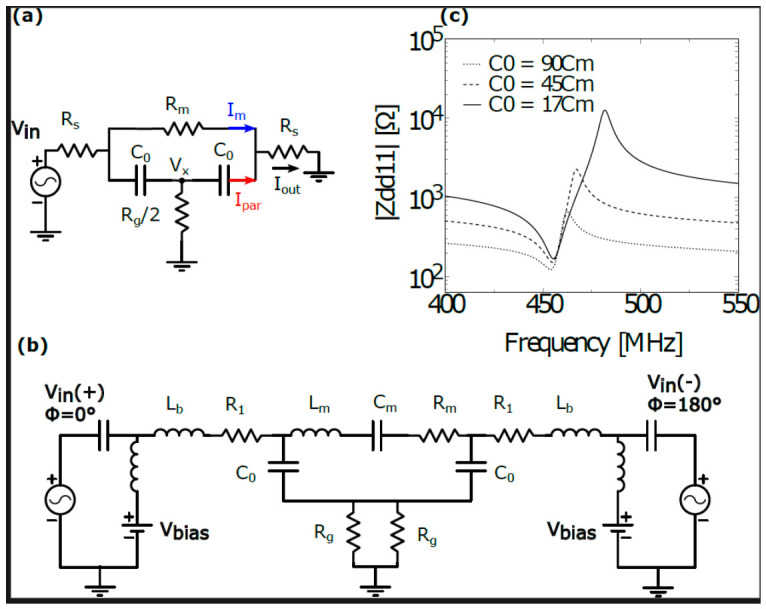
(**a**) A 2-port SAW resonator lumped model, including motional inductance, $L_{m}$, motional capacitance, $C_{m}$, motional resistance, $R_{m}$, static capacitance, $C_{0}$, and grounding resistance, $R_{g}$. (**b**) Simplified configuration of a conventional 2-port SAW resonator around the resonance frequency. (**c**) Extended lumped model for a 2-port SAW resonator including the bond wire inductance, $L_{b}$, metal resistance, $R_{1}$, and differential actuation.

The size of the resonant cavity cannot be too large, especially the transmitting IDTs, because of the large parasitic feed-through as a result of the high dielectric constant of PZT. The effect of the parasitic capacitance on the impedance response can be analyzed using a lumped element model of the resonator. When the resonator is operated close to its resonant frequency, the lumped element model simplifies to a motional resistor $R_{m}$ in parallel with the parasitic capacitance $C_{0}$, as shown in Figure 2a. If $C_{0}R_{g}\omega_{0}<<1$, the current through $C_{0}$, the parasitic path $I_{\mathrm{par}}$ (see Figure 2a), has approximately 180° phase difference, relative to the current through the motional branch $I_{m}$ of the resonator at resonance. Here, *R*_g_ is the grounding resistance. This is due to the high-pass filtering between $V_{x}$ (the bottom electrode voltage) and $V_{\mathrm{in}}$ (the input voltage) formed by $C_{0}$ and $R_{g}$. The phase of $I_{\mathrm{par}}$ is 90° larger than the phase of $V_{x}$ and is therefore out of phase with $V_{\mathrm{in}}$. The current, $I_{m}$, is in phase with $V_{\mathrm{in}}$; therefore, $I_{\mathrm{par}}$ and $I_{m}$ are out of phase by 180°. Consequently, this leads to a reduction in the total current delivered to the measurement load resistor, $R_{s}$, which shows the importance of minimizing $C_{0}$. In order to mitigate the influence of $R_{g}$ differential actuation is used by applying two signals which are 180° out of phase to the two input IDTs $V_{\mathrm{in}}\left(+\right)$ and $V_{\mathrm{in}}\left(-\right)$. Based on the lumped model of the SAW resonator [15,22], shown in Figure 2b, the mixed-mode input impedance can be determined from the two port impedance parameters, $Z_{ij}=V_{i}/I_{j}{|}_{I_{k}=0,k\neq j}$, and expressed under the assumption of symmetry as:

(1) $$\begin{array}{l} Z_{\mathrm{dd}11}=\\ \frac{\tau \left(s^{2}+\omega_{0}^{2}\right)+\frac{s}{\omega_{B}}\left(s^{2}+\omega_{0}^{2}\right)+s+\omega_{0}^{2}s^{-1}+\frac{\omega_{0}}{Q}\left(1+\tau s+\frac{s^{2}}{\omega_{B}}\right)}{C_{0}\left(s^{2}+\omega_{0}^{2}+\frac{\omega_{0}}{Q}s\right)}, \end{array}$$

where s=jω, τ, Q, $\omega_{B}$ and $\omega_{0}$ are, respectively, $R_{1}C_{0}$, $\omega_{0}L_{m}/R_{m}$, $1/\sqrt{L_{b}C_{0}}$ and $1/\sqrt{L_{m}C_{m}}$. Here, *L*_m_ and *C*_m_ are the motional inductance and capacitance, respectively, *L*_b_ is the bond wire inductance, and *R*_1_ is the metal resistance. From the differential impedance model, it is observed that it does not depend on the ground resistance, and the high-pass filtering effect is therefore mitigated by differential actuation. The effect of $C_{0}$ on the differential impedance is shown in Figure 2c. It is observed that, for increasing capacitance, the impedance magnitude becomes smaller; also, the spacing between series and parallel resonance peak, defined by the minimum and maximum impedance, respectively, becomes smaller. The results of the impedance model are compared with the measurements in Section 4.

## 3. Device Fabrication and Characterization

**Figure 3:**
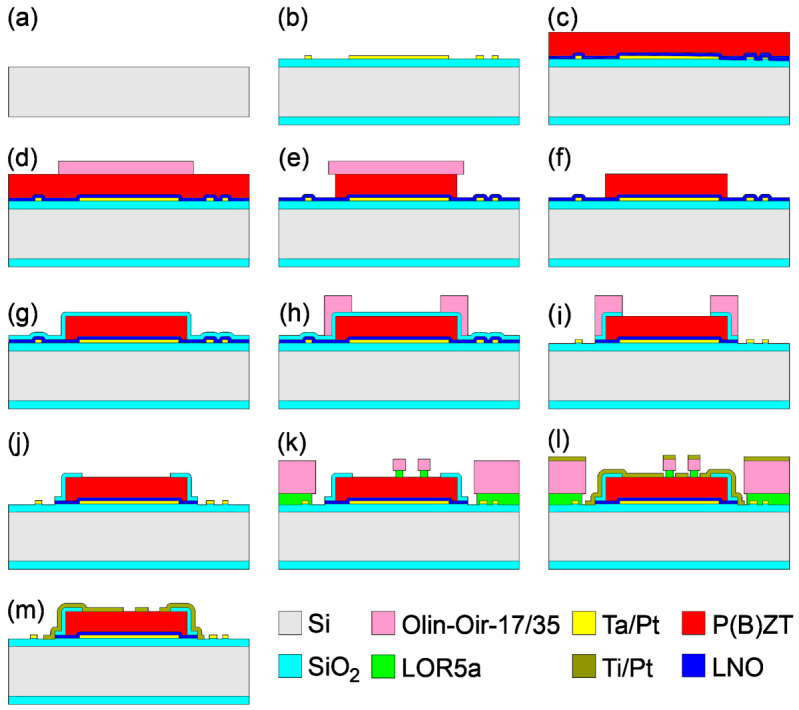
Cross-sectional overview, perpendicular to the wave propagation direction, of the SAW resonator fabrication: (**a**) DSP Si wafer; (**b**) patterning resist for lift-off, deposition of Ta/Pt bottom electrode and lift-off; (**c**) PLD of LNO seed layer and P(B)ZT; (**d**) patterning resist and (**e**) wet chemical etching of P(B)ZT; (**f**) strip resist; (**g**) PECVD SiO_2_ capping layer deposition; (**h**) patterning resist followed by (**i**) directional etching of SiO_2_ capping layer and LNO seed layer; (**j**) strip resist and (**k**) pattern next resist layer stack for lift-off top electrode; (**l**) deposition of Ti/Pt top electrode material and (**m**) lift-off.

**Figure 4:**
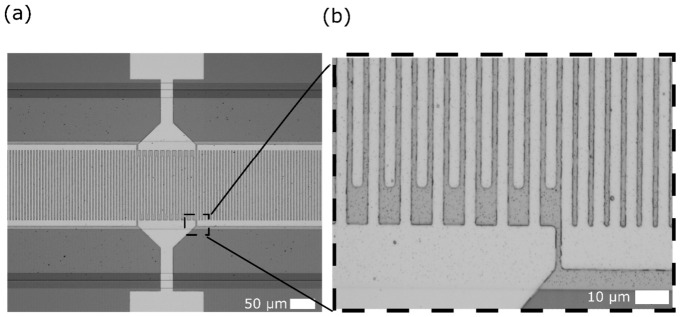
(**a**) SAW resonator with λ=8 μm. (**b**) Zoom in on the IDT and grating reflectors.

A series of SAW resonators were fabricated for different λ of 16, 12 and 8 μm. All devices consist of five IDT pairs and 75 grating reflectors placed on both sides of the IDTs. This IDT amount was chosen to keep C_0_ small while having sufficient actuation and detection of the desired mode, which is possible due to the high *K*^2^. As discussed in the previous section, the aperture of the IDTs of all devices is equal to 10λ. A schematic representation of the four-mask fabrication process is shown in Figure 3. The SAW devices are manufactured on Double-Side Polished Si(100) p-type wafers with a thickness of 525 μm and an electric resistivity of 5–10 Ωcm (see Figure 3a). First, a 500 nm thermal silicon-dioxide (t-SiO_2_) layer is grown by means of wet oxidation at 1150 °C. Lift-off resist is used for the deposition of the bottom electrodes. For this, LOR-5a and Olin 907-17 photoresist are spin-coated on top of the t-SiO_2_ layer and are patterned using the first mask. A layer of 10/150 nm Ta/Pt is RF-magnetron-sputtered, and the lift-off is performed in an ultrasonic bath with acetone. Subsequently the LOR-5a resist is removed with Olin OPD-4262 developer, Figure 3b. As adhesion layer for Pt, Ta is used instead of Ti because of the better stability at high temperatures during the PLD step [23]. A (001)-dominant P(B)ZT film on top of a 10 nm LaNiO_3_ (LNO) seeding layer is deposited using a SolMateS PLD system (see Figure 3c). The Ba content was 10 mol-% in all cases, similar to previous PLD work by one of the co-authors [24]. In total, three different P(B)ZT layer thicknesses are deposited: 750, 1000 and 1600 nm. The films were prepared at 600 °C substrate temperature with an oxygen pressure of 0.1 mbar. The P(B)ZT film is patterned by wet chemical etching (see Figure 3e) using the second mask (see Figure 3d). By removing the P(B)ZT outside the resonant cavity, the top metal can be deposited onto SiO_2_ layer, preventing delamination during wire bonding. After resist stripping, Figure 3f the P(B)ZT layer is capped with a SiO_2_ layer of 100 nm deposited by means of Plasma Enhanced Chemical Vapor Deposition (see Figure 3g). The third mask is used to pattern the SiO_2_ capping layer (see Figure 3h), and is etched using Reactive Ion Etching (see Figure 3i); thus, after resist stripping, only the P(B)ZT sidewalls are covered (see Figure 3j). Dielectric capping is necessary in order to prevent Ohmic contact between the top metallization and the LNO seeding layer, which is exposed after wet etching of the P(B)ZT film. Contact between the top metal and LNO results in a finite resistance between the signal input and ground, limiting the resonator performance. For the top electrodes, the lift-off process is used once more with 10/150 nm Ti/Pt patterned using the fourth mask, Figure 3k–m. The frontside of the wafer is covered with Olin 908-35 photoresist for protection during stripping of the backside SiO_2_. The backside SiO_2_ is etched using buffered hydrofluoric acid. Finally, the wafer is diced and the frontside photoresist is removed with acetone and cleaned with isopropanol. A top-view microscope image of a fabricated SAW device is shown in Figure 4.

**Figure 5:**
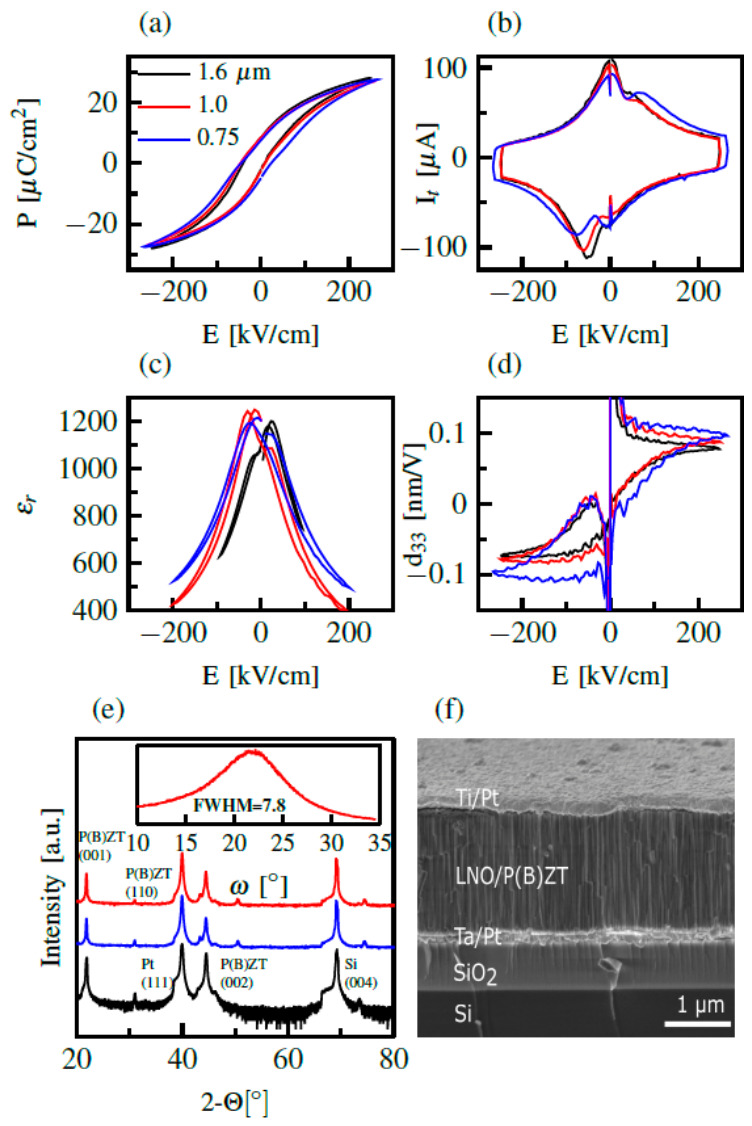
Measured material properties: (**a**) polarization loops; (**b**) switching current; (**c**) dielectric constant; (**d**) piezoelectric coefficient; (**e**) XRD pattern, θ–2θ scan, of the P(B)ZT films the inset shows the (002) rocking curve; (**f**) SEM cross-section.

A metal insulator metal 200 × 200 μm capacitor is used for measuring the ferro- and piezoelectric material properties of the P(B)ZT film. Doping is typically used for altering the ferro- and piezoelectric properties of the PZT layer. Doping PZT with Ba lowers the dielectric constant while a higher electric breakdown field is obtained, at the expense of a small decrease in the piezoelectric coefficients. The polarization hysteresis loop (P-E) measurement, Figure 5a, was performed using a ferroelectric analyzer using a triangular ac-electric field of ±250 kV/cm at 1 kHz. The remnant and saturation polarization equal 8.5 μC/cm^2^ and 28.4 μC/cm^2^, respectively, and the coercive field strength equals 8 kV/cm. These values are somewhat lower than measured values in other work in which PLD was used to deposit Ba-doped PZT [25].

Two peaks are visible in the switching current in Figure 5b, one at the high-electric fields and one at the zero-electric field. The former is associated with switching of the ferroelectric domains, while the latter is associated with the switching of non-ferroelectric domains. The fact that both are present is attributed to the poly-crystalline structure of the film. It is known that, if the P(B)ZT layer is leaky due to defects such as grain boundaries, the ferroelectric response can be suppressed [26]. The difference in ferroelectric behavior between layers therefore most likely results from the difference in structural properties. A possible solution for this is exposing the layer to a high number of domain-switching cycles which gradually lowers the leakage current. Figure 5c shows the dielectric constant of the layers, with high values at E = 0 and lower values for E = ±200 kV/cm. The maximum value of ca. 1200 compares very well with the highest values in other work in the field of PLD [24,25,27,28]. The out-of-plane deformation of the capacitor is measured by means of a double-beam laser interferometer with a dc-field strength of ±250 kV/cm and an ac-field amplitude of 5 kV/cm at 1 kHz. The largest amplitudes measured are 2, 2.2 and 3 nm, for h = 0.75, 1.0 and 1.6 μm, respectively, and result from the larger applied dc-bias voltage. The piezoelectric coefficient d_33_, Figure 5d, is computed from the measured amplitude and the maximum is equal to d_33_ = 120 pm/V. This value is high compared to published values for Ba-doped PZT films deposited by PLD [25,27] but similar to previous work on the same system [24]. The large ‘overshoots’ arise from polarization switching from antiparallel to parallel to the applied electric field, inducing a large increase in the strain (in the out-of-plane direction, accompanied by a large increase in the stress in the in-plane directions), while the domain structure does not adapt immediately to the new in-plane stress state [29]. To prevent the formation of pyrochlore phases at the interface of the P(B)ZT film and the Pt layer a 10 nm LNO nucleation layer is deposited before P(B)ZT deposition. The crystallinity of the piezoelectric stack is determined by means of X-ray diffraction (XRD) (see Figure 5e). The XRD shows that the P(B)ZT films on Pt are predominantly (001)-oriented with a small fraction of (110)-orientation. No evidence of other crystalline phases is found. The full width at half maximum (FWHM) value of the rocking curves of the P(B)ZT (002) peak equals 7.8°. This relatively high value is the result of the Pt sheet layer that prevents a higher preferred orientation. Lower values might be desired for SAW applications, but nevertheless, due to the large $K^{2}$ of the P(B)ZT film, quite a good performance is still achieved. The cross-sectional SEM image, Figure 5f, shows the expected columnar growth of the film. The initial nucleation of the P(B)ZT growth layer has a significant effect on the growth of the grains and is determined for a large part by the granular structure of the LNO layer deposited on Pt, which promotes columnar P(B)ZT growth. As the results show though, the density of the columns is high enough for SAW propagation.

## 4. Results and Discussion

The SAW resonators are characterized using a Rohde & Schwarz ZVB-20 Vector Network Analyzer; to this purpose, the chips are mounted on a four-layered Printed Circuit Board (PCB) with a total thickness of 1 mm and FR-4 as a dielectric material. The SAW resonator chip is wire bonded with gold wires to the PCB. The PCB traces are designed as a coplanar waveguide with a ground plane with a characteristic impedance of 50 Ω. The desired modes are actuated by applying a differential signal between the two IDT ports. The differential S-parameters are determined from the two port S-parameters according to, $S_{\mathrm{dd}11}=0.5\left(S_{11}+S_{22}-S_{12}-S_{21}\right)$. All measurements have been performed at 0 dBm input power and a Short-Open-Load-Thru calibration is performed. The dc-bias voltage is applied to the input of a Bias-T using a Delta Electronika power supply and connected to the IDTs. The measurement circuit is shown schematically in Figure 2.

**Figure 6:**
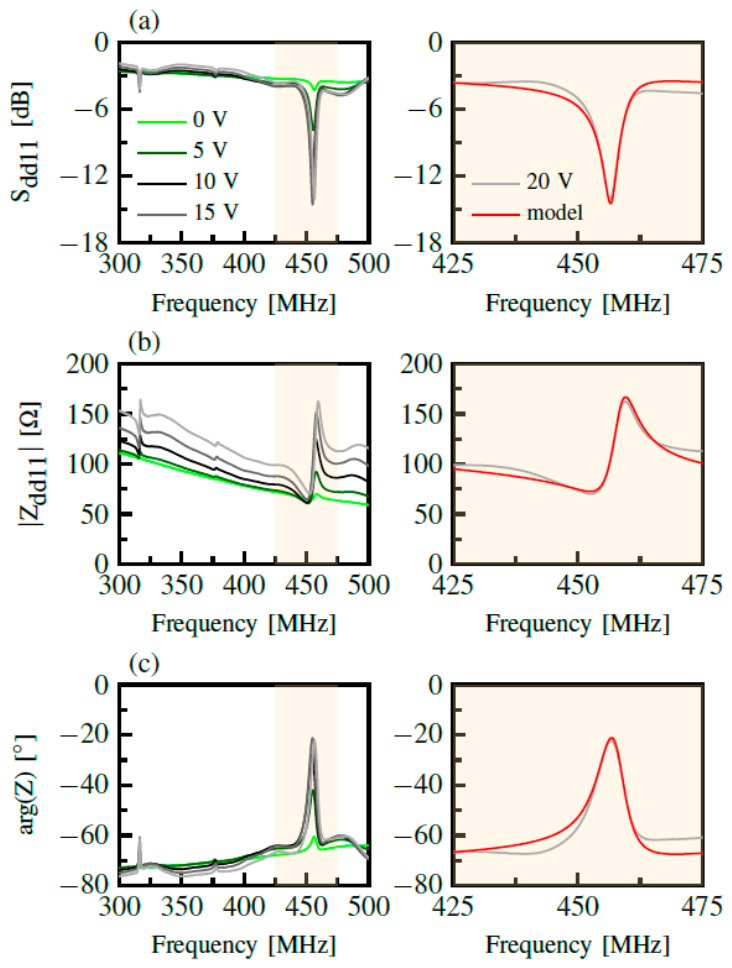
Measured frequency response with h = 1.6 μm and λ= 8 μm. (**a**) S_dd11_, (**b**) impedance and (**c**) phase. The zoomed-in frequency range compares the measured data with the theoretical model.

**Figure 7:**
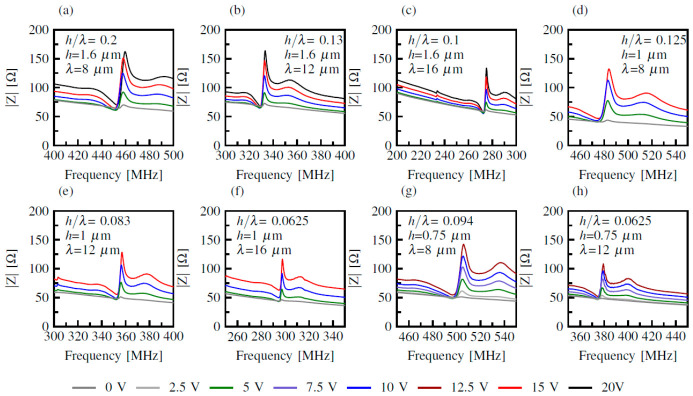
Impedance measurement results: (**a**) h = 1.6 μm, λ= 8 μm; (**b**) h = 1.6 μm, λ= 12 μm; (**c**) h = 1.6 μm, λ= 16 μm; (**d**) h = 1.0 μm, λ= 8 μm; (**e**) h = 1.0 μm, λ= 12 μm; (**f**) h = 1.0 μm, λ= 16 μm; (**g**) h = 0.75 μm, λ= 8 μm; (**h**) h = 0.75 μm, λ= 12 μm.

The measured frequency response of a fabricated device with h = 1600 nm and λ=8 μm is shown in Figure 6. Two resonant modes are present at two distinct frequencies at approximately 315 MHz and 450 MHz and their mode shape can be identified based on the $V_{p}$ and their occurrence in the measured frequency spectrum. The lower frequency is the Rayleigh mode because this corresponds to the fundamental mode with a $V_{p}$ of 2520 m/s. The $V_{p}$ is larger than that of a bulk PZT substrate because the layers underneath the P(B)ZT also contribute to the effective shear modulus. The higher frequency is the Sezawa mode because this is the first higher-order mode. This is what is expected based on the simulation results as shown in Figure 1. Furthermore, it is observed that, for increasing the dc-bias voltage from 0 V to 20 V, the S_dd11_, impedance, and phase response are increasing. This is because $C_{0}$ is decreasing while the piezoelectric coefficient is increasing at a higher dc-bias voltage, as shown in Figure 5c,d. The increase in the piezoelectric coefficient is due to the reorientation of the piezoelectric domains. At low dc-bias voltage (0–10 V), the main contribution is due to domain reversal, while at higher voltages (15–20 V), most of the switchable domains already have been aligned. At high dc-bias voltages, the resonators operates more efficiently, but the corresponding decrease in shear modulus will lead to a shift in the resonant frequency of ∼2 MHz. The measured impedances of the Sezawa mode for different ratios of h/λ, extracted from the differential S-parameters, are shown in Figure 7. For decreasing layer thickness and increasing wavelength it is observed that the magnitude of the impedance is decreasing. This indicates that the electromechanical conversion, in combination with the large feedthrough capacitance, is not sufficient to maintain the impedance amplitude response for these h/λ ratios.

**Figure 8:**
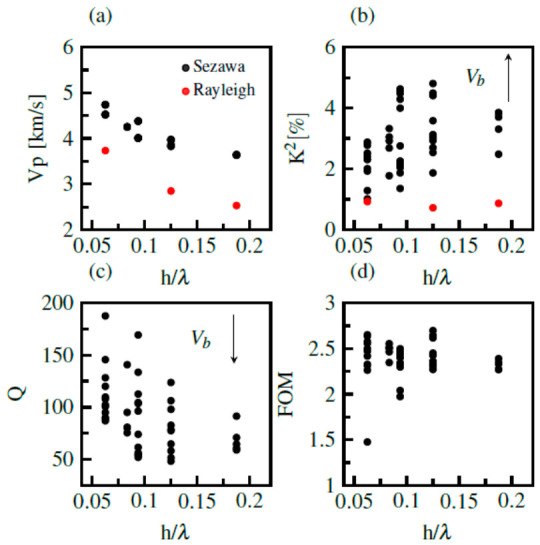
Calculated resonator performance parameters (**a**) phase velocity, (**b**) electromechanical coupling factor, (**c**) Q-factor, and (**d**) FOM factor. Arrow direction indicates increase in dc-bias voltage.

To quantify the performance of the SAW resonator, the values for $V_{p}$ and $K^{2}$ are determined from the measurement data according to:

(2) $$\begin{array}{rr} V_{p}= & \left(f_{s}+f_{p}\right)\lambda /2, \end{array}$$

(3) $$\begin{array}{rr} K^{2}= & \left(\pi f_{s}/2f_{p}\right)/tan\left(\pi f_{s}/2f_{p}\right), \end{array}$$

where $f_{s}$ and $f_{p}$ are the series and parallel resonant frequency, respectively. The dispersion curves for $V_{p}$ and $K^{2}$ are shown in Figure 8a,b up to a normalized thickness of 0.2. From Figure 8a, it is observed that the measured $V_{p}$ is larger for the Sezawa mode than for the Rayleigh mode, values up to 4800 m/s are obtained which is an increase compared to single-crystalline piezoelectric substrates. Similar observations hold for the $K^{2}$ values in Figure 8b indicating that the Sezawa mode is superior to the Rayleigh in terms of $V_{p}$ and $K^{2}$. This is what is expected: when h/λ<0.2 the Sezawa mode is dominant, whereas, for h/λ>0.2, the Rayleigh mode becomes dominant [30]. The maximum obtained values for $K^{2}$ at h/λ = 0.12 are comparable with those of single-crystalline piezoelectric substrates. It is observed that for increasing dc-bias voltage $K^{2}$ is increasing. This is because the ratio $f_{s}/f_{p}$ decreases indicating that the frequency bandwidth determined by $f_{s}$ and $f_{p}$ is increasing at higher dc-bias voltages, as shown in Figure 7a–h. This is expected since $C_{0}$ decreases for increasing dc-bias voltage. However, for h/λ<0.08, the $K^{2}$ in combination with the large $C_{0}$ is not sufficient to maintain the impedance response.

There are many methods for determining the Q-factor [31,32]. However, for resonators with a low Q-factor, the measurement precision suffers from unwanted spectral components as a result of parasitic effects. This results in a rotation and translation away from the origin in the complex plane of the measured coefficients. In order to separate these effects from the ideal second-order characteristics of the resonator, a circle fit is performed and is rotated such that the resonant frequency is on the positive real axis [32,33]. The phase angle is then computed with respect to the positive real axis and the phase as a function of frequency (see Equation (4)), and is fit to this by using nonlinear least squares [32,33].

(4) $$\begin{array}{r} \phi \left(f\right)=\phi_{0}-2arctan\left(Q\left(\frac{f}{f_{0}}-\frac{f_{0}}{f}\right)\right), \end{array}$$

where $\phi_{0}$ is the phase angle at resonance and $f_{0}$ is the resonant frequency. The obtained Q-values for the Sezawa mode are shown in Figure 8c. The resonators show Q-values below 200. The effect of the dc-bias voltage on the Q-factor is opposite to that of the $K^{2}$. For an increase in dc-bias voltage, the Q-factor becomes lower. Seemingly counter intuitive, this can be understood from second order differential equation of motion for mechanical systems. The Q-factor is proportional to the ratio of effective stiffness to damping. From the results in Figure 7, it can be observed that, with an increase in dc-bias, the resonant frequency becomes smaller, indicating a lower shear modulus. It is reasonable to assume that the damping is not significantly affected by the dc-bias since the effective resistance of the resonators is significantly reduced as a result of an increase in the $K^{2}$. The decrease in the Q-factor is therefore mainly due to the decrease in stiffness. Increasing h/λ also lowers the Q-factor, which is expected since the damping increases due to a larger contribution of the P(B)ZT layer. For resonator applications there is a trade-off between the Q and the $K^{2}$ values; therefore, a figure of merit (FOM) can be defined which is equal to the product of Q and $K^{2}$. The FOM as function of h/λ is shown in Figure 8d and remains approximately constant for the various layer thicknesses and dc-bias. The $K^{2}$ can be further increased by reducing the parasitic path, $C_{0}$ [14,15], improving the structural density of the PZT. Since patterning of the bottom electrode to reduce feed-through capacitance between the two ports primarily affects performance under single-ended actuation, $C_{0}$ is mainly determined by the layer thickness and the IDT design. The columnar microstructures present in the deposited PZT layer introduce mechanical discontinuities that dissipate acoustic energy. A denser PZT is expected to increase both the conversion efficiency and Q-factor, as the PZT layer will be uniform, with few voids or grain boundaries. This allows the acoustic wave to propagate smoothly with less scattering and friction. Furthermore, dense films typically exhibit more homogeneous polarization, resulting in stronger electromechanical coupling. A higher $V_{p}$ can be targeted by thinning of the t-SiO_2_ layer.

The performance compared to other multilayered material systems for Sezawa SAW resonators is listed in Table 1. It is observed that the $K^{2}$ values for P(B)ZT on Si are among the highest. The Q-factor is slightly lower than the other material systems, but in combination with the large $K^{2}$, this results in similar FOM values as for the Si/GaN and sapphire/AlN multilayers. Compared with SiC and sapphire substrates, silicon is more attractive for certain MEMS applications—such as sensors and actuators; this is because it supports well-established and well-understood micromachining processes. This maturity enables the fabrication of complex device structures and designs. The primary material systems for comparison are therefore the Si/GaN [34] and the Si/AlScN [35] multilayers. As with the Si/ALScN multilayer, the P(B)ZT film on Si exhibits a low h/λ, which allows easier integration with microfabrication processes and provides better control over the piezoelectric film quality. While the Si/ALScN system performs better in terms of FOM, it should be noted that, for the (B)PZT layer in the current work, the PLD material “design space” has not yet been fully explored, and improvements can be expected from a more optimized crystal domain orientation and possibly from a denser layer.

## 5. Conclusions

In conclusion, (001)-oriented P(B)ZT films were deposited on bulk Si and two port SAW resonators were fabricated. Differential actuation of the SAW resonators effectively mitigated the influence of $R_{g}$. Both Rayleigh and Sezawa modes were successfully characterized within the 275–500 MHz frequency range, with the Sezawa mode demonstrating higher values of $K^{2}$ and $V_{p}$. Reorientation of the piezoelectric domains using a dc-bias voltage across the P(B)ZT film resulted in a significant increase between 200 and 300% in the $K^{2}$ values. The Q-factor decreases due to the reduction in effective stiffness; however, its order of magnitude remains essentially unchanged. Consequently, the FOM remains approximately constant over the considered h/λ range. This work demonstrates that P(B)ZT is a promising material for RF-MEMS applications that require high $K^{2}$, as it effectively excites the Sezawa mode even at h/λ<0.2, while also offering the potential for resonator performance tuning. However, more work is needed to improve the Q-factor since this is currently limiting the overall performance. This can possibly be achieved by optimizing the dopant and using different PLD grow templates, e.g., seed layers (${\mathrm{CeO}}_{2}$ and YSZ), such that the structural density and crystal quality of PZT can be improved. From a device design perspective, the performance of the resonator can be improved by optimizing the acoustic-resonant cavity using trenches in combination with metal reflectors [38]. This will result in higher SAW reflections upon each discontinuity, resulting in more acoustic confinement inside the resonant cavity. The results presented here serve as a first experimental step toward scalable, PZT-on-silicon SAW systems. Beyond this initial demonstration, further enhancements are envisioned through careful design optimization and material engineering.

## Figures and Tables

**Table 1:** Comparison with prior art.

Material	Mode	*f* [GHz]	h/λ	Q	$K^{2}$ [%]	FOM
Si/GaN [34]	1-port	6–8	1.2–2.1	100–150	1.5–3.5	2.25–3.5 ^a^
SiC/V:ZnO [6]	1-port	4–6	0.15–0.65	450–600	3–5	16–24
SiC/ZnO [6,7]	1-port	5–7	0.15–0.65	450–750	1.5–2.5	9–16
Sapphire/AlN [36]	1-port	0.43	0.24	7000	0.03	2.1
Si/AlScN [35]	1-port	0.31	0.05	230–300	4.1	10
SiC, Sapphire/Al(BSc)N [37]	2-port	4.3–7.6	0.4–0.6	150–1400	0.4–4.0	1–22
Si/P(B)ZT ^b^	2-port (diff)	0.3–0.5	0.06–0.2	50–200	1–5	1.5–2.75

^a^ N/A estimated from provided Q and $K^{2}$ values. ^b^ This work.

## Data Availability

The data generated for and used in this article will be made available by the authors on request.
